# Antimicrobial activity of a selection of organic acids, their salts and essential oils against swine enteropathogenic bacteria

**DOI:** 10.1186/s40813-019-0139-4

**Published:** 2019-12-27

**Authors:** Manuel Gómez-García, Cinta Sol, Pedro J. G. de Nova, Mónica Puyalto, Luis Mesas, Héctor Puente, Óscar Mencía-Ares, Rubén Miranda, Héctor Argüello, Pedro Rubio, Ana Carvajal

**Affiliations:** 1 Department of Animal Health, Faculty of Veterinary Medicine, Universidad de León, Campus de Vegazana, s/n, 24007 León, Spain; 2 Norel SA, Calle Jesús Aprendiz n° 19, 28007 Madrid, Spain

**Keywords:** Pig, feed additives, organic acids, Essential oils, Minimum inhibitory concentration, Minimum bactericidal concentration, Fractional inhibitory concentration, enteric pathogens

## Abstract

**Background:**

Accurate screening of new alternative antimicrobial compounds is essential for their use to control pathogens in swine production due to the replacement of antibiotics and zinc oxide. Most in vitro studies have separately reported the antimicrobial activity of organic acids and essential oils (EOs) using diverse methods for susceptibility testing. In addition, in vitro outcomes can help in the selection of the suitable antimicrobial compound and effective combinations of these compounds in the control of pathogens of interest in pork production. Therefore, the aim of this study is to determinate the antibacterial activity of six organic acids and six EOs against *Escherichia coli*, *Salmonella* spp. and *Clostridium perfringens* isolates, some of them multi-resistant to antibiotics, from swine origin. The synergistic effects between the products with higher activity for each bacteria were also calculated.

**Results:**

All products tested showed activity against at least one bacterial species, except for black pepper EO. The results showed that formic acid with the shortest chain length was the most effective against *E. coli* and *Salmonella* spp.*,* while the sodium salt of coconut fatty acid distillates with long chain acids was the most effective against *C. perfringens*. The susceptibility of isolates tested to EOs was similar, a result that demonstrates a similar activity of these products against phylogenetically unrelated pathogens. In addition, an additive effect was shown for carvacrol-oregano EO for *E. coli*, formic acid-carvacrol and formic acid-thymol for *Salmonella* spp. and carvacrol-cinamaldehyde for *C. perfringens*.

**Conclusions:**

The susceptibility of isolates to EOs was similar, a result that demonstrates a similar activity of these products against phylogenetically unrelated pathogens in contrast to organic acids. In addition, an additive effect was shown for several combinations of these compounds.

## Background

Nowadays, demand for antibiotic-reduced or antibiotic-free farm animals is rising globally. Its achievement can only be accomplished through a combination of strategies, which include husbandry, biosecurity and alternatives to antibiotics [1, 2]. The swine industry is foremost in antimicrobial consumption [3]. Consequently, pig enteric pathogens frequently harbour antimicrobial resistance to a large number of compounds. As there is a requirement to reduce the use of antibiotics, other compounds with antimicrobial activity could be considered to replace them within control strategies. In this context, the use of plant extracts or phytobiotics and organic acids have gained renewed interest because of their potential antimicrobial activity [1, 4–7]. These compounds can be used as feed additives, potentially conferring benefits to health and growth to the host due to their antimicrobial activity and immune response enhancement [4, 8].

Essential oils (EOs) are volatile lipophilic compounds constituted of a complex mixture of terpenoids and phenols [9, 10] and are one of the most interesting groups of phytobiotic compounds [11, 12]. Their antibacterial activity seems to be associated with the disruption of the structure and function of bacteria cell membranes due to their hydrophobicity [12]. Organic acids have also antimicrobial properties based on their ability to cross the cell membrane, due to the lipophilic nature of their undissociated form, modifying the proton and associated anion concentrations in the cytoplasm [13]. Consequently, purine bases and essential enzymes are negatively affected and bacterial viability decreases [14]. These acids are generally available as calcium, potassium or sodium salts to decrease odour and volatility and facilitate the manufacturing processes [15]. Despite their well-known antimicrobial properties, field results are not always succesful [16–18]. These results demonstrate that further research is needed in order to optimise concentrations, combinations and interactions of these compounds against target pathogens. This information would be considered of value to increase the accuracy of treatments with EOs and/or organic acids.

The present study evaluates the antimicrobial activity of six organic acids and six EOs against *E. coli*, *C. perfringens* and *Salmonella* spp. Interactions between the compounds with greater activity against each bacterial species were also investigated.

## Methods

### Bacterial strains and growth conditions

The eighteen strains used in this study belonged to the collection of the Infectious Diseases Unit (IDUC) at the Veterinary Faculty of the University of León. The collection included strains from the Spanish Type Culture Collection (CECT) and field isolates recovered from faecal samples from diarrhoea outbreaks on Spanish swine farms. Table 1 summarises the main characteristics of strains used, including virotype for *E. coli*, serotype for *Salmonella* spp. and toxigenic type for *C. perfringens*. In addition, the antimicrobial resistance profile of each strain was determined by disc diffusion test, using breakpoint values provided by Clinical and Laboratory Standard Institute [19] and the Comité de l’Antibiogramme de la Société Française de Microbiologie.

**Table 1:** Main characteristics of bacterial strains used

*Escherichia coli*	Virotype	Antibiotic resistance profile
EC 60 ^a^	STb	CEF, LIN
EC 61 ^a^	STb	LIN
EC 67 ^a^	F18, STa, STb, EAST1	LIN, ENR, SUL
EC 96 ^a^	STb	AML, CEF, LIN, SUL
EC 107 ^a^	–	AML, CEF, LIN, SUL, DOX
EC 115 ^a^	F18, EAST1	AML, LIN, SUL
*Salmonella* spp.	Serotype/Phagotype	Antibiotic resistance profile
SP 11 ^a^	Typhimurium/DT 104	AML, SPC, LIN, SUL, FFC
SP 28 ^a^	London	LIN, SUL
CECT 443	Typhimurium	LIN
CECT 700	Infantis	AML, CEF, LIN, SUL, DOX
CECT 915	Choleraesuis	LIN
CECT 4300	Enteriditis	LIN, SUL
*Clostridium perfringens*	Toxigenic type	Antibiotic resistance profile
CP 3 ^a^	Type A (alpha toxin)	CEF, LIN, ENR, SUL
CP 34 ^a^	Type A (alpha toxin)	SUL
CP 52 ^a^	Type A (alpha toxin)	LIN, ENR, SUL
CP 89 ^a^	Type A (alpha toxin)	Not detected
CP 99 ^a^	Type A (alpha toxin)	LIN, SUL
CP H ^a^	Type A (alpha toxin)	SUL

*AML* amoxicillina, *SPC* spectinomycin, *ENR* enrofloxacin, *SUL* sulphanomides, *DOX* doxycycline, *CEF* cephalotin, *LIN* lincomycin, *FFC* florfenicol

^a ^Field isolates recovered from faecal samples collected from swine farms in Spain

Frozen cultures stored at − 80 °C were revived by inoculation onto appropriate agar plates, tryptic soy agar (TSA) (Scharlab, Spain) for *E. coli* and *Salmonella* spp. and fastidious anaerobe agar (FAA) (Neogen, United Kingdom) for *C. perfringens. E. coli* and *Salmonella* isolates were incubated at 37 °C for 24 h under aerobic conditions while *C. perfringens* were grown at 38.5 °C for 24 to 36 h in an anaerobic workstation with an oxygen-free anaerobic gas mixture (80% N_2,_ 10% H_2_ and 10% CO_2_). Subsequently, the purity of the cultures was confirmed for each strain by examination of colony morphology and Gram staining.

### Organic acids and essential oils

The products evaluated in this study were provided by Norel SA (Spain). They included six organic acids: formic acid (purity 85%), propionic acid (99%), sodium butyrate (98%), sodium heptanoate (95%), pelargonic acid (99%) and sodium salt of coconut fatty acid distillates (67%), and six EOs: cinnamaldehyde (97–98%), thymol (99%), carvacrol (99%), clove EO (eugenol 80%), oregano EO (phenols 65–75%) and black pepper EO (piperine 40%). Formaldehyde (40%) was also tested as a positive control.

The organic acids sodium butyrate, sodium heptanoate and sodium salt of coconut fatty acid distillates were obtained as powder products and were resuspended in 50 mM sodium phosphate buffer (pH 6.0) depending on their solubility. Final concentrations of these stock solutions were 500,000 ppm (w/v) for sodium butyrate, 50,000 ppm (w/v) for sodium heptanoate and 10,000 ppm (w/v) for the sodium salt of coconut fatty acid distillates. Other organic acids and formaldehyde were provided as liquid preparations and two stock solutions were prepared at 38400 ppm (v/v) for each product in Mueller-Hinton broth (Cultimed, Spain) and brain heart infusion (BHI) (Merck, Germany). Finally, EOs were diluted 1:1 in sterile propylene glycol (Sigma-Aldrich, United States) and then in the same broth media to obtain a final concentration of 38,400 ppm (v/v) as well. All the stock solutions were stored at room temperature.

### Antibacterial activity

#### Inoculum preparation

For each tested isolate, three or four fresh bacterial colonies (after 24 to 36 h incubation) were suspended in a NaCl solution (0.85% w/v) to achieve a turbidity between 1 and 2 on the McFarland scale. Before 15 min, these bacterial suspensions were diluted 1:1000 in Mueller-Hinton broth for *E. coli* and *Salmonella* spp. and BHI for *C. perfringens.* Both broth media were previously adjusted to pH 6.0.

#### Determination of minimum inhibitory concentration (MIC)

Susceptibility testing was carried out using the broth microdilution method previously described [5], in sterile flat bottom 96-well microplates (Jet Biofil, Canada) with a final bacterial concentration of approximately 10^5^ CFU/mL and a volume of 200 μL per well (100 μL of diluted product and 100 μL of bacterial suspension).

Previously, for each assay, the products were diluted in the specific broth media adjusted to pH 6.0 to obtain double the initial concentration tested for each bacterial species. The initial concentration range for each product was determined, taking into account the recommended inclusion rate in feed provided by the supplier. Hence, the inclusion rates in feed recommended in animal production are 1000 ppm for sodium butyrate, 3000 ppm for sodium heptanoate and sodium salt of coconut fatty acid distillates, 250 ppm for the rest of organic acids and 150 ppm for all evaluated EOs. However, if no growth inhibition was detected within the initial range tested, concentrations were increased until inhibition was observed or the maximum possible concentration of the product was reached. The final concentration range evaluated for each product against the three bacterial species is summarised in Table 2.

**Table 2:** Final concentration range (ppm) evaluated for each product and bacterial species tested

	Concentration (ppm)					
	*E. coli*		*Salmonella* spp.		*C. perfringens*	
	Maximum	Minimum	Maximum	Minimum	Maximum	Minimum
Formic acid	2400.0	37.5	2400.0	37.5	4800.0	75.0
Propionic acid	9600.0	75.0	4800.0	37.5	4800.0	75.0
Sodium butyrate	250,000.0	390.6	250,000.0	390.6	250,000.0	390.6
Sodium heptanoate	25,000.0	390.6	25,000.0	390.6	25,000.0	390.6
Pelargonic acid	19,200.0	300.0	38,400.0	37.5	4800.0	75.0
Sodium salt of coconut fatty acid distillates	5000.0	78.1	5000.0	78.1	2500.0	2.0
Cinnamaldehyde	2400.0	75.0	2400.0	75.0	2400.0	75.0
Thymol	2400.0	75.0	2400.0	75.0	2400.0	75.0
Carvacrol	2400.0	75.0	2400.0	75.0	2400.0	75.0
Clove EO	2400.0	75.0	2400.0	75.0	2400.0	75.0
Oregano EO	2400.0	75.0	2400.0	75.0	2400.0	75.0
Black pepper EO	250,000.0	75.0	250,000.0	75.0	250,000.0	75.0
Formaldehyde	4800.0	4.7	1200.0	4.7	4800.0	9.4

Incubation was carried out under shaking at 2 x g and 37 °C for 24 h for *E. coli* and *Salmonella* spp. or at 38.5 °C for 24 to 36 h in an anaerobic box (Oxoid, United States) using anaerobic generator sachets (Anaerogen, Oxoid, United States) for *C. perfringens*. All susceptibility tests were performed in triplicate and a maximum difference of one two-fold dilution in MIC values between replicates was allowed.

The MIC was established as the first concentration without visible growth. Data were used to establish the lowest concentration of each product, which inhibited the growth of 50% (MIC_50_) of the strains for each bacterial specie included in the study.

### Determination of minimum bactericidal concentration (MBC)

Bactericidal tests were performed as have been previously described [5], using TSA for *E. coli* and *Salmonella* spp. and FAA for *C. perfringens*. Plates were incubated under optimal conditions for the growth of each bacterial species and the assays were performed in triplicate. The MBC value was defined as the lowest concentration of a product which kills 99.9% or more of the bacteria in the original inoculum (less than 5 colonies). Median (MBC_50_) of the MBC was estimated for each bacterial species.

### Determination of fractional inhibitory concentration index (FICI)

For each evaluated bacterial species, the four products with lower MIC_50_ (two EOs, one organic acid and positive control formaldehyde) were selected to investigate their interactions in pair-wise combinations. Combined drug antibacterial activity was assessed by means of the FICI and using a modified microdilution checkerboard method as previously described by other authors [20]. For each bacterial species, a randomly selected strain was employed for this determination (*E. coli* EC 61, *Salmonella* spp. CECT 4300 and *C. perfringens* CP 34). The assays were designed in order to allow the determination of the MIC value of each product alone as well as the combination-derived MIC simultaneously and were carried out in 96-well microplates with a final volume of 200 μL per well. Briefly, within each microplate, the first six rows were used to study the combined effect of the products with six different concentrations being evaluated for each product, including the previously determined specific MIC_50_. Rows G and H include positive and negative control wells and dilutions of each tested product alone for the estimation of the MIC for each single product. Conditions for incubation were the same as previously used for MIC determination and each assay was performed in triplicate.

FICI for each combination was obtained using the following formula:

$$ FICI= FI{C}_{product\;A}+{\mathrm{FIC}}_{\mathrm{product}\;\mathrm{B},} $$

where FIC_product A_ = MIC_A in combination_/MIC_A alone_ and FIC_product B_ = MIC_B in combination_/MIC_B alone_.

Finally, combinations were classified as synergistic (FICI ≤0.5), additive (FICI > 0.5 and ≤ 1), indifferent (FICI > 1 and ≤ 4) or antagonistic (FICI > 4) in accordance with Odds [21].

## Results

### Minimum inhibitory concentration (MIC)

Tables 3, 4, 5 and 6 summarise the MICs for each evaluated product against the three pathogens tested. All tested products, with the only exception of black pepper EO, decreased the growth of at least one of the evaluated strains. In most cases, the differences of MIC values were not greater than one two-fold dilution among the strains of the three bacterial specie, showing intra-species homogeneous susceptibility.

**Table 3:** Susceptibility of the strains included to the organic acids tested and formaldehyde

Strain	MIC (ppm)						
	Formic acid	Propionic acid	Sodium butyrate	Sodium heptanoate	Pelargonic acid	Sodium salt of coconut fatty acid distillates	Formaldehyde
EC 60	600.0	2400.0	62,500.0	3125.0	4800.0	> 5000.0	150.0
EC 61	600.0	1200.0	62,500.0	3125.0	4800.0	> 5000.0	150.0
EC 67	600.0	2400.0	62,500.0	3125.0	4800.0	> 5000.0	150.0
EC 96	1200.0	1200.0	50,000.0	781.3	2400.0	> 5000.0	150.0
EC 107	600.0	1200.0	50,000.0	781.3	4800.0	> 5000.0	150.0
EC 115	600.0	1200.0	50,000.0	1562.5	2400.0	> 5000.0	150.0
SP 11	600.0	1200.0	125,000.0	3125.0	19,200.0	> 5000.0	75.0
SP 28	600.0	1200.0	125,000.0	781.3	4800.0	> 5000.0	75.0
CECT 443	600.0	1200.0	125,000.0	1562.5	4800.0	> 5000.0	75.0
CECT 700	600.0	1200.0	125,000.0	781.3	19,200.0	> 5000.0	75.0
CECT 915	600.0	1200.0	125,000.0	1562.5	4800.0	> 5000.0	75.0
CECT 4300	600.0	1200.0	125,000.0	1562.5	4800.0	> 5000.0	75.0
CP 3	2400.0	4800.0	31,250.0	3125.0	2400.0	32.0	75.0
CP 34	1200.0	2400.0	31,250.0	3125.0	1200.0	16.0	75.0
CP 52	1200.0	2400.0	62,500.0	1562.5	1200.0	16.0	150.0
CP 89	2400.0	4800.0	31,250.0	1562.5	2400.0	16.0	150.0
CP 99	1200.0	2400.0	31,250.0	3125.0	2400.0	16.0	75.0
CP H	2400.0	4800.0	31,250.0	1562.5	2400.0	8.0	150.0

**Table 4:** Susceptibility of the strains included to the essential oils tested

Strain	MIC (ppm)					
	Cinnamaldehyde	Thymol	Carvacrol	Clove EO	Oregano EO	Black pepper EO
EC 60	600.0	1200.0	300.0	600.0	600.0	> 250,000.0
EC 61	300.0	600.0	300.0	300.0	300.0	> 250,000.0
EC 67	600.0	600.0	600.0	600.0	600.0	> 250,000.0
EC 96	600.0	600.0	150.0	600.0	300.0	> 250,000.0
EC 107	600.0	1200.0	600.0	600.0	1200.0	> 250,000.0
EC 115	600.0	600.0	300.0	300.0	300.0	> 250,000.0
SP 11	600.0	600.0	300.0	600.0	600.0	> 250,000.0
SP 28	300.0	1200.0	600.0	600.0	1200.0	> 250,000.0
CECT 443	300.0	300.0	300.0	600.0	600.0	> 250,000.0
CECT 700	600.0	300.0	300.0	600.0	600.0	> 250,000.0
CECT 915	600.0	300.0	300.0	600.0	600.0	> 250,000.0
CECT 4300	600.0	300.0	300.0	600.0	600.0	> 250,000.0
CP 3	300.0	600.0	300.0	1200.0	600.0	> 250,000.0
CP 34	150.0	1200.0	300.0	600.0	300.0	> 250,000.0
CP 52	300.0	1200.0	300.0	1200.0	300.0	> 250,000.0
CP 89	300.0	1200.0	300.0	600.0	600.0	> 250,000.0
CP 99	300.0	1200.0	300.0	600.0	600.0	> 250,000.0
CP H	150.0	1200.0	600.0	600.0	600.0	> 250,000.0

**Table 5:** MIC_50_, MBC_50_ and MBC_50_/MIC_50_ ratio of the organic acids tested and formaldehyde

		Formic acid	Propionic acid	Sodium butyrate	Sodium heptanoate	Pelargonic acid	Sodium salt of coconut fatty acid distillates	Formaldehyde
*E. coli*	MIC_50_	600.0	1200.0	50,000.0	1562.5	4800.0	> 5000.0	150.0
	MBC_50_	2400.0	9600.0	125,000.0	1562.5	19,200.0		150.0
	MBC_50_/MIC_50_	4.0	8.0	2.5	1.0	4.0	Not applicable	1.0
*Salmonella* spp.	MIC_50_	600.0	1200.0	125,000.0	1562.5	4800.0	> 5000.0	75.0
	MBC_50_	2400.0	2400.0	125,000.0	1562.5	19,200.0		300.0
	MBC_50_/MIC_50_	4.0	2.0	1.0	1.0	4.0	Not applicable	4.0
*C. perfringens*	MIC_50_	1200.0	2400.0	31,250.0	1562.5	2400.0	16.0	75.0
	MBC_50_	2400.0	2400.0	62,500.0	3125.0	2400.0	16.0	150.0
	MBC_50_/MIC_50_	2.0	1.0	2.0	2.0	1.0	Not applicable	2.0

**Table 6:** MIC_50_, MBC_50_ and MBC_50_/MIC_50_ ratio of the EOs tested

		Cinnamaldehyde	Thymol	Carvacrol	Clove EO	Oregano EO	Black pepper EO
*E. coli*	MIC_50_	600.0	600.0	300.0	600.0	300.0	> 250,000.0
	MBC_50_	1200.0	1200.0	300.0	600.0	600.0	
	MBC_50_/MIC_50_	2.0	2.0	1.0	1.0	2.0	Not applicable
*Salmonella* spp.	MIC_50_	600.0	300.0	300.0	600.0	600.0	> 250,000.0
	MBC_50_	2400.0	600.0	600.0	1200.0	600.0	
	MBC_50_/MIC_50_	4.0	2.0	2.0	2.0	1.0	Not applicable
*C. perfringens*	MIC_50_	300.0	1200.0	300.0	600.0	600.0	> 250,000.0
	MBC_50_	300.0	1200.0	300.0	1200.0	600.0	
	MBC_50_/MIC_50_	1.0	1.0	1.0	2.0	1.0	Not applicable

Among organic acids, the best results were obtained with formic acid against *E. coli* and *Salmonella* spp. (MIC_50_ 600 ppm for both bacterial species). Sodium salt of coconut fatty acid distillates inhibited the growth of *C. perfringens* at very low concentrations (MIC_50_ 16 ppm) but showed no activity against Gram negative bacteria (MIC_50_ > 5000 ppm). Our results revealed a similar susceptibility of all the strains to the tested EOs with black pepper EO being the only exception, with MIC_50_ values ranging between 300 and 1200 ppm. In detail, minimum MIC_50_ (300 ppm) was found for carvacrol and oregano EO against *E. coli*, carvacrol and thymol against *Salmonella* spp. and carvacrol and cinnamaldehyde against *C. perfringens.* Finally, the three bacterial species were susceptible to very low concentrations of formaldehyde with MIC_50_ ranging from between 75 and 150 ppm.

### Minimum bactericidal concentration (MBC)

MBC values varied among the three tested bacterial species (Tables 5 and 6). Sodium salt of coconut fatty acid distillates showed a pronounced bactericidal activity against *C. perfringens* (MBC_50_ 16 ppm). Pelargonic acid also had a higher bactericidal activity against *C. perfringens* (MBC_50_ 2400 ppm) as compared to Gram negative bacteria (MBC_50_ 19,200 ppm).

MBC_50_/MIC_50_ ratio (Tables 5 and 6) revealed that the highest differences between bacteriostatic and bactericidal concentrations were found among Gram negative bacteria exposed to formic acid, pelargonic acid, propionic acid, cinnamaldehyde or formaldehyde.

### Fractional inhibitory concentration index (FICI)

The lowest FICI values for each combination and strain are listed in Table 7. None of the combinations showed synergism for any of the evaluated bacterial species. Nevertheless, an additive interaction was observed for all the tested combinations, with the only exception being formaldehyde-formic acid and formaldehyde-oregano EO against *E. coli*, with FICI values 1.06 and 1.13, respectively. The combinations with lower FICI values were formaldehyde-carvacrol and carvacrol-oregano EO for *E. coli* (0.63), formic acid-carvacrol and formic acid-thymol for *Salmonella* spp. (0.56) and carvacrol-cinamaldehyde for *C. perfringens* (0.63).

**Table 7:** FICI obtained for the combined antimicrobial products

*E. coli*			*Salmonella* spp.			*C. perfringens*		
Combination of products		FICI	Combination of products		FICI	Combination of products		FICI
Formaldehyde	Formic acid	1.06	Formaldehyde	Formic acid	1.00	Formaldehyde	Sodium salt of coconut fatty acid distillates	1.00
	Carvacrol	0.63		Carvacrol	1.00		Carvacrol	1.00
	Oregano EO	1.13		Thymol	0.75		Cinnamaldehyde	0.75
Formic acid	Carvacrol	0.75	Formic acid	Carvacrol	0.56	Sodium salt of coconut fatty acid distillates	Carvacrol	1.00
	Oregano EO	1.00		Thymol	0.56		Cinnamaldehyde	1.00
Carvacrol	Oregano EO	0.63	Carvacrol	Thymol	1.00	Carvacrol	Cinnamaldehyde	0.63

## Discussion

A number of studies have proposed that organic acids and phytobiotics as the EOs are an interesting alternative to antibiotics use in human and veterinary medicine as well as into carcass decontamination strategies, due to their recognized antibacterial activity [4, 5, 9, 22]. Controversy about antibacterial activity (i.e. MIC concentration) reported in different studies could be explained by differences in technical aspects of the methods used for the estimation of this activity [23] and bacterial species or strains included in the studies [24]. Here, we have focused on the activity of organic acids and EOs against three significant swine enteropathogenic bacteria (*E. coli*, *Salmonella* spp. and *C. perfringens*). The study determines with accuracy the most appropriate products and the potential necessary inclusion rate against each of these three enteric pathogens, providing knowledge for future field research regarding efficacy.

The results of our study confirmed the antibacterial effect of all products tested against the three bacterial species except for black pepper EO. This result is in contrast to previous studies, which have reported activity of this compound against *E. coli* [25]. The absence of antimicrobial activity of black pepper EO in our study could be a consequence of the low concentration of piperine (40%) since piperine has been proposed as the major active principle in black pepper EO and is responsible for its main properties [26].

Interestingly, differences in susceptibility of the strains included for each pathogen were scarce, showing a relative low range of MIC. However, the assessment of antimicrobial activity of these products might help to establish the specific dosage for particular strains detected either on farm or at post-harvest. Short-chain organic acids showed higher effect on *Enterobacteriaceae* bacteria, while medium chain organic acids seemed to have higher activity against *C. perfringens*. The outer membrane of Gram-negative bacteria makes the passage of medium size or large molecules more difficult, in contrast to Gram-positive bacteria, which do not possess this membrane. Additionally, protein channels embedded into the lipid bilayer only allow the diffusion into the cytoplasm of Gram-negative bacteria of low molecular weight compounds [27, 28]. Accordingly, formic acid with the shortest chain length showed the highest antibacterial activity against *E. coli* and *Salmonella* spp. (MIC_50_ 600 ppm for both species) followed by propionic acid. The sodium salt of coconut fatty acid distillates, which main active component is lauric acid, was the most effective against *C. perfringens* (MIC_50_ 16 ppm) but was not capable of inhibiting the growth of *E. coli* and *Salmonella* spp. at the concentrations tested (MIC_50_ > 5000 ppm). Organic acids could be considered as bioactive compounds when their MIC is equal or lower than 1000 ppm [29, 30]*.* From our results, only formic acid was shown to be active (MIC 600–1200 ppm) against both *E. coli* and *Salmonella* spp. and the sodium salt of coconut fatty acid for *C****.*** *perfringens*. As demonstrated in this study, current in feed inclusion rates of these products are generally lower than MIC_50_ values. On one hand, these results highlight the relevance of in vitro assays to determine the concentrations at which feed additives exert their antimicrobial effect against enteric pathogens. On the other, compounds such as butyric acid have demonstrated indirect activity, inhibiting the expression of virulence factors required to infect the host [31], fact that can explain why sub-bacteriostatic concentrations of these compounds can have beneficial effects on the animal health and performance. In addition, results of clinical trial are also influenced by technical aspects and biological factors, which can modify the composition and activity of the products. Bioactivity of all tested EOs was similar between Gram-negative and Gram-positive bacteria as previously described [32]. With the only exception of thymol against *C. perfringens*, all the EOs evaluated can be considered as antimicrobials since their MIC_50_ values are in the range of 100 to 1000 ppm in accordance with other authors [33]. In addition, the bioactivity of thymol and oregano EO against *E. coli,* thymol and carvacrol against *Salmonella* spp. and cinnamaldehyde and carvacrol against *C. perfringens* can be classified as moderate (MIC range 126 to 500 ppm), being mild (MIC range 501 to 1000 ppm) for the rest of the EOs [29, 30].

With regard to bactericidal activity, MBC_50_ values were equal or slightly higher than MIC_50_ values for most of the products tested against the three bacterial species. Similar results have been obtained in previous reports with carvacrol and thymol against the three bacterial species [34] as well as with eugenol, cinnamaldehyde, thymol and carvacrol against *E. coli* [35]. The MBC_50_/MIC_50_ ratio in all products that achieved antimicrobial activity was equal or less than 4 and, consequently, these products could be classified as potentially bactericidal agents according to previous proposed cut-off values [36], with the only exception of propionic acid for *E. coli*. This last product, whose MBC_50_/MIC_50_ ratio was 8, could be considered as a bacteriostatic against *E. coli*.

In agreement with our FICI findings, an additive effect has been reported for several binary combinations of EOs against different bacterial species [34, 35, 37] or for combinations of EOs and organic acids [24]. These additive effects would allow the use of binary combinations of products in feed. In this way, a recent research has confirmed the benefits on growth performance of weaned piglets of dietary supplementation with a mixture of organic acids (fumaric acid, citric acid, sorbic acid and malic acid) and EOs (cinnamaldehyde and thymol) [38].

## Conclusions

The results obtained allow us to conclude that most of the organic acids and EOs evaluated have in vitro bactericidal activity against these three enteric pathogens. Essential oils exhibited a broad spectrum while based on our results we recommend short chain organic acids to control *E. coli* and *Salmonella* spp. and sodium salt of coconut fatty acid for *C. perfringens* infections. The additive effect showed for several combinations of these compounds points at the interest of using combinations of these antimicrobials in infection treatments and strategies to reduce the risk of transmission of these pathogens in meat processing plants.

## Data Availability

The authors declare that they do not apply new software and databases.

## Abbreviations

BHI: Brain heart infusion
CECT: Spanish Type Culture Collection
EO: Essential oil
FAA: Fastidious anaerobe agar
FICI: Fractional inhibitory concentration index
IDUC: Infectious Diseases Unit
MBC: Minimum bactericidal concentration
MIC: Minimum inhibitory concentration
TSA: Tryptic soy agar
